# Cultural influences behind cholera transmission in the Far North Region, Republic of Cameroon: a field experience and implications for operational level planning of interventions

**DOI:** 10.11604/pamj.2017.28.311.13860

**Published:** 2017-12-15

**Authors:** Moise Chi Ngwa, Alyson Young, Song Liang, Jason Blackburn, Arabi Mouhaman, John Glenn Morris

**Affiliations:** 1 Department of International Health, Johns Hopkins Bloomberg School of Public Health, Baltimore, Maryland, USA; 2 Institute for Child Health Policy, Texas EQRO, Department of Health Outcomes and Policy, College of Medicine, University of Florida, Gainesville, Florida, USA; 3 Emerging Pathogens Institute, University of Florida, Gainesville, Florida, USA; 4 Department of Environmental and Global Health, College of Public Health and Health Professions, University of Florida, Gainesville, Florida, USA; 5 Spatial Epidemiology and Ecology Research Laboratory, Department of Geography, College of Liberal Arts and Sciences, University of Florida, Gainesville, Florida, USA; 6 Department of Environmental Sciences, Higher Institute of the Sahel, University of Maroua, Cameroon; 7 Department of Medicine, College of Medicine, University of Florida, Gainesville, Florida, USA

**Keywords:** Culture, cholera, etiology, spread, practices, Far North, Cameroon

## Abstract

**Introduction:**

In recent years, the Far North Region of Cameroon has experienced serious and recurrent cholera outbreaks. Yet, understanding of cultural influences on outbreaks and spread remain poorly understood. This qualitative study explored cultural influences on cholera exposure in this region.

**Methods:**

Interviews and group discussions were conducted in two phases. Phase I involved key informants and phase II included focus group and household discussions. Thematic techniques including word repetition, key-indigenous-terms, and key-words-in-context were used for qualitative data analysis.

**Results:**

Key informants attributed cholera etiology to dirt and spread through water (*caneri*) and food (group eating or *faire-un-rond*) while group discussions attributed it to a reprimand from *god* and transmission through the air. Participants suggested that funerals, weddings, open defecation, and mountaintop burial might influence cholera exposure and facilitate its spread. Hospital avoidance and non-adherence with cholera treatment regimens were linked to favorable beliefs about traditional medicine (rural-urban mentality confrontation). Furthermore, a multiplicity of ethnic languages, mistrust of message sources, culture of dependency and sentimental animal husbandry were barriers to the reception of public health messages.

**Conclusion:**

Many participants had limited scientific knowledge about cholera etiology and transmission. The cultural practice of mountain burial seemed to explain the high cholera attack rate in the mountainous terrain compared to the floodplains. Cultural factors are likely to play important roles in the exposure to and spread of cholera. Understanding cultural context, individual and community perceptions of risk and disease may help public health agencies in response to outbreak prevention and control.

## Introduction

**Figure 1:**
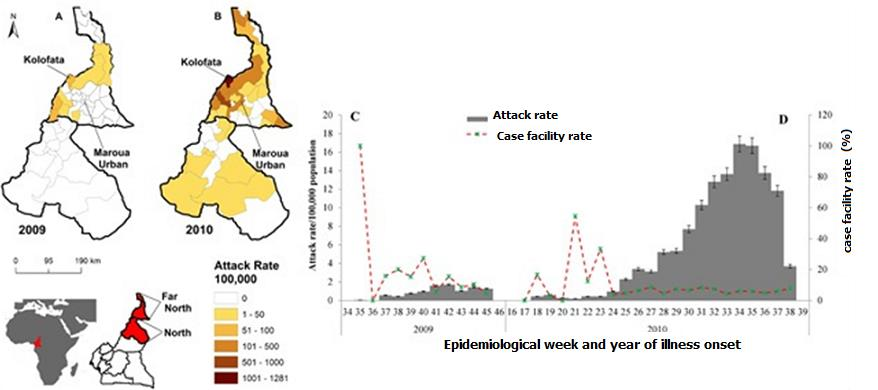
Spatial (A and B) and temporal (C and D) distribution of cholera attack rate per 100,000 inhabitants in the North and Far North Regions, Cameroon, 2009-2010. In 2009, cholera was limited in the Far North (A) and (C); in 2010, it started in the North and spread to the Far North (B) and (D); the case fatality ratio (red dashed line of C and D) far exceeded 50% at the start of the epidemic in both years.

**Figure 2:**
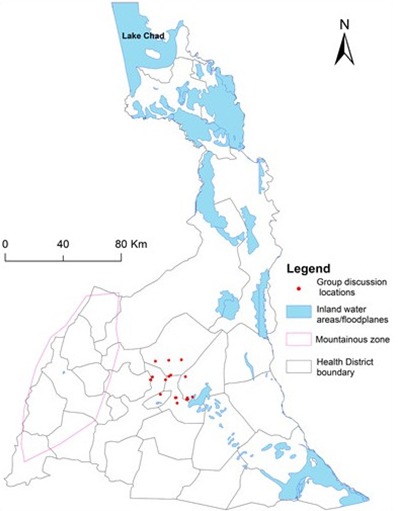
Locations of focused group discussion and house hold discussions (red dots) during phase II of the study; the map shows Far North is a diverse terrain. The western portion is mountainous (purple line) while the eastern portion contains the Logon and Chari floodplains (blue color); reported cholera cases are higher in the mountainous terrain than in the floodplains terrain

The ongoing 7^th^ cholera pandemic's [1, 2] true burden is estimated at 1.3 to 4.0 million cases yearly, and 21,000 to143, 000 deaths worldwide [3, 4]. This pandemic hit the continent of Africa in 1970 [5, 6] and Cameroon in 1971 in Goulfey [7-11] in the Far North (FN) of the country (Figure 1 A-D). In 2009, the worst outbreak in Cameroon since 1971 started in the FN, and Kolofata Health District, located in mountainous terrain and border with Nigeria (Figure 1 A, C), was hit the hardest. Another outbreak began in the FN in 2010, and Kolofata (Figure 1 B, D) again reported the highest cases. Yet, 80% of cholera cases can easily be treated with oral rehydration salts [12]. During 2009/2010, cholera, a prototypical waterborne disease, affected health districts of the western mountainous terrain far more than districts in the eastern Logon/Chari floodplains of the FN (Figure 2). This was counter intuitive, raising questions as to whether cultural practices and beliefs influenced the cholera transmission.

The FN is one of the most culturally diverse regions in Cameroon with more than 50 ethnic groups, including the Fulbe, Mandara, Kapsiki, and Kanouri [13]. Literature shows that culture, ‘a system of shared beliefs, values, customs, behaviors, and artifacts’ [14], has the potential to influence infectious disease transmission [15,16], including cholera [17-19]. For instance, the study of Louis et al. in Guinea reported that people who attended a rural funeral were more affected by the outbreak that ensued than those who did not [20]. However, despite the cultural diversity in the FN, we found only one publication that explored knowledge, attitude and social representations with regard to cholera [21]. Thus, gaps exist in research on cultural influences on cholera transmission in the FN. This study explored how cultural practices and beliefs influence cholera transmission in the FN, Cameroon. The study is of vital significance because data obtained from it will have utility in generating hypotheses that may be tested in subsequent studies. At a more immediate level, an understanding of cultural influences on cholera exposure is important for informing interventions for reducing cholera transmission.

## Methods

**Setting:** The settings of this study were Maroua Urban Health District (an urban health district located in the FN Capital, Maroua), and Kolofata Health District (a rural health district) (Figure 1, Figure 2) located about 79 Km from Maroua. Kolofata was of interest because it borders Nigeria and had the highest attack rates (500 and 1282 cases per 100,000 inhabitants) during 2009 and 2010 outbreaks, respectively (Figure 1 A and B). These health districts were chosen, firstly, because of our existing contacts in the field. Secondly, we wanted to explore whether cultural influences on cholera transmission differed in the rural and urban settings.

**Study design, sampling and participants:** This study used an exploratory cross-sectional (qualitative) study design. Participants were recruited using purposive and snowball sampling strategies [22] in two phases. Phase I focused on key informants (KIs) who were required to be literate while phase II centered on grassroots laypeople in the community who were not required to be literate. These two phases were designed to capture whether literate KIs and grassroot laypersons have a shared understanding of cultural influences on cholera. In phase I, a purposeful sample of KI participants was initially selected from the University of Maroua, and then extended outward to include workers in the Regional Public Health Delegation, cholera command and control center, District Hospitals, and traditional rulers. The sample was not random but did aim for representativeness. In both the rural and urban settings, fieldwork was conducted from February to March 2015 in English and French, the two official languages in Cameroon. Questions and hypotheses coming from phase I were explored in phase II.

In phase II, in urban settings, a purposeful sample of grassroot laypersons was selected through door-to-door recruitment and placed into groups of 9 to 12 participants each; sampling was not done in rural settings because of security concerns associated with Boko Haram. The first group was formed with help from Community Health Workers (CHWs) and subsequent groups were constituted using a snowball strategy [22]. The compositions of the ten groups were stratified by age (18-30, 31-60, and >60) and gender with four women's groups, four men's groups, and two of both sexes. The latter group was included to explore whether both sexes might complement each other as they discuss cultural aspects of cholera. In addition, we also sampled seven households, having a mean of four family members, led by the head of household. The seven households resulted from relationships that were built during group formation and were conducted with view of not omitting vital information that could emerge from the households. Fieldwork was conducted from April to July 2015.

**Data collection:** Research methods incorporating key informant interviews (KIIs), focus group discussions (FGDs), and household discussion (HHDs) were used to collect data in the two phases, respectively. Both KIIs (phase I) and FGDs/HHDs (phase II) were guided using open-ended questions about perceptions and beliefs about the etiology of cholera, as well as cultural practices and behaviors that may influence individual's exposure to cholera, receptivity or ability to act on public health information against cholera. The duration of KIIs ranged from thirty minutes to one-and-half hours and from two to three hours for FGDs in French and English. During FGDs/HHDS, participants were encouraged to expresses their views in their own vocabulary, and explore further themes of interest to them. Field notes and interviews were recorded on notebooks and audio (voice) recorders. Figure 2 shows the locations of the FGDs during phase II study. The data were encrypted and stored at Emerging Pathogens Institute's (EPI's) secure InfoPath database for analysis.

**Dealing with local languages:** in phase I, which focused on KIs, all participants were fluent in French or English or both; and thus, local language transcription and translation was not needed. However, during FGDs/HHDs in phase II, local language issues become apparent. Nevertheless, making use of knowledge from phase I, the interview team consisted of two students and one researcher from the University of Maroua (UM) and one CHW all with excellent fluency in the local languages. During FGDs/HHDs, one UM student read the open-ended questions in french, the CHW translated into local language, and translated back into French as the participants spoke. The other UM student took field notes in French and UM researcher audio typed the conversations and confirmed that what was translated reflected what the participants said. At the end of each discussion, what was said in local language was already in French in both paper and audio formats. The transcriptions were then focused on the French version.

**Data analysis:** Tape-recorded interviews, discussions, and field notes in French were transcribed and translated into English. The English versions were back translated into French to check whether the original meaning was captured during translation into English. In the transcriptions, important aspects of data interpretation such as voice speed, tone, and points of stress were captured. The transcripts were arranged according to KI and FGDs/HHDs data. The qualitative data analysis was inductive to ensure that the process focused on data. Focus groups and individual interviews were sorted and coded hierarchically into themes incorporating techniques including word repetition, key-indigenous terms, and key-words-in-context [23]. Data coding were performed using pen and paper. Computer software was not used. Some text fit into more than one theme or code. In working through the transcripts, sections of text from KIs, FGDs, and HHDs that related to a theme or code were brought together. This was to ensure that the sources of the various texts could be re-traced. Thus, thematic similarities and dissimilarities of cultural influences on cholera transmission could easily be inferred.

**Ethical Considerations:** IRB from the University of Florida (IRB201500123), Cameroon Ministry of Public Health (No D21-15/MF/MINSANTE/SG/DLM/CSE), Regional Public Health Delegation for the FN (No 103/L/15/MINSANTE/SG/DRSPEN/SISP/ham), and Regional Center for Research and Innovation for the FN (N/Ref. 01/MINSI/CRRI-EN/SAG/2015) approved this qualitative study prior to data collection in all phases. Participants' consent were obtained prior to interviews/discussions. No information with personal identifiers was obtained.

## Results

The following broad themes including: 1) cultural beliefs of cause and perceptions of spread of cholera, 2) cultural practices and behaviors that influence exposure to cholera, and 3) cultural traditions that influence how people receive public health information for the fight against cholera.

### Beliefs about etiology and perceptions of cholera spread

**Figure 3:**
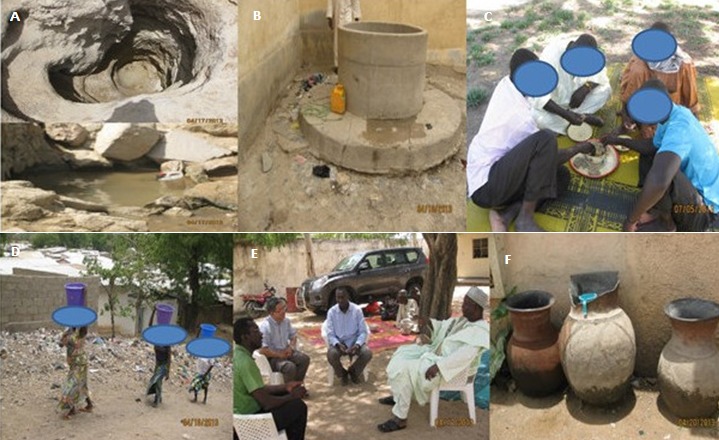
Some environmental conditions and cultural aspects that influence cholera exposure transmission: The community sources water from A potholes (top), ponds (bottom), and B open wells (everyone uses the yellow container in B to draw water), hallmarks of risky water sources. Group eating out of the same bowl using fingers C is a culture that can aid rapid cholera transmission and spread while water is transported D in open-mounthed containers, as explained by our Key Informant E (Key Informant—right, interviewers—left), and stored in open-mouthed jars F called ‘caneri’, placed at strategic locations from which all can dip water using a common cup (blue cup in the middle jar of F). Water driven travel (contact between folks from mountain and valley) in D could underlie rapid disease transmission. The Figure shows the environment, which together with culture, shape behavior that leads to cholera exposure.

**Causal beliefs:** in exploring cultural beliefs of cause and spread of cholera, KIs identified cholera as caused by dirt linked with water and food (Figure 3 A-C). *‘Cholera is caused by and is a disease of dirt linked with water and food’* (KI, traditional chief, Kolofata). They explained disease etiology in terms of fetching water from high-risk sources such as ponds, ditches, and open wells (Figure 3 A and B). In contrast, FGDs/HHDs attributed cholera etiology to the supernatural. *‘Cholera is caused by punishment from ‘god’.* (FGD and HHD, women only group, Doualare, Maroua II). The cause of cholera as punishment from ‘god’ blames the patient for failing to please ‘god’. This is a typical notion that leads many villagers to consult soothsayers who will direct them to perform rituals such as offering food to ‘god’ (commonly known in the local language as *‘sadaka’*) to prevent and/or cure cholera; and thus, delaying seeking proper care at health facilities. This stands in stark contrast to the understanding of cholera as a waterborne disease seen with the KIs.

### Perception of spread

***Cholera spreads through air:*** Some villages infer that cholera spreads through the air when they said, *‘cholera spreads mainly through the air’* (HHD, Doulare, Maroua II). As stated previously, this constitutes a perception that leads villagers to consult soothsayers who will direct the type of rituals to be performed to prevent the spread of cholera, which delays seeking proper care at health facilities.

***Communal water usage:*** KIs and FGDs alike identified communal water usage, open-mouthed jars placed at convenient locations where all can dip water with a single cup for drinking (Figure 3 D-F), as a factor that influences rapid cholera spread: *the culture is such that the community shares water and so communal shared water usage (‘L'utilisation communautaire de l'eau’) contributes to the rapid spread of cholera because folks dip the same cup in open-mouthed pots* (KI, traditional chief, Kolofata).

KIs mentioned communal water usage, but did not use its common local languages name *‘caneri’* (Figure 3 F). The name *‘caneri’* (was first mentioned during FGDs/HHDs, whose participants recognized the potential for water contamination by dipping, but felt impossible to avoid using it. *We cannot avoid the ‘caneri’. For us, it is a natural refrigerator. Yes, during cholera period, the risk of contaminating water in ‘caneri’ is very high. However, there is no such discussion as avoiding the ‘caneri’-No way! It has been handed to us by previous generations, and even with the threat of cholera, there is nothing like avoiding it* (HHD, Domayo, Maroua I).

The above excerpt points to communal water usage (henceforth *‘caneri’*) as a natural refrigerator given by God, which has been handed down by previous generations as participants put it. These two factors explain the strong emotional attachment to ‘caneri’. Although water storage in ‘caneri‘is risky for disease spread, even the threat of cholera will not discourage its use in the present form in the Far North.

**Group eating:** Both KIs and FGDs perceived group eating with hands out of same bowl using fingers (Figure 3 C) as a risk factor for cholera spread but differed as to whether it could be avoided. With FGDs, prohibition of group eating was a categorical no as the excerpt illustrates: *Group eating is a cultural way of life linked with feeling of belonging to a family. Rather, people should be encouraged to make a round (‘faire un rond’),* (FGD, men and women groups, Doualare, Maroua II).

The phrase make a round (*‘faire un rond’*) is understood to mean eating together out of the same bowl with hands using fingers (Figure 3 C), which is a cultural way of life symbolizing solidarity and camaraderie. Clearly, group eating with fingers is a cholera risk factor. However, in the FN it represents belonging to a family, which should be encouraged. Apparently, people understand the biomedical arguments about cholera transmission, but there are cultural and practical reasons why they continue with traditional practices.

### Cultural practices that influence cholera exposure

**Funerals:** All participants agreed that funeral practices expose attendees who eat at such gathering at heightened risk of cholera. The key issue about funerals for KIs was eating at funerals: *Culturally, there is high population travel (‘mobilité culturelle’) for funeral and practices, which exposes massive sections of the population at risk of cholera at a given time. As such, travels to eat at funerals constitute rapid means of cholera exposure between villages,*(KI, traditional chief, Kolofata).

What is termed population travel or *‘mobilité culturelle’* refers to people who come in contact when visiting a household to attend a funeral with such gatherings bringing people together thereby creating opportunities for cholera exposure and eventual transmission. Eating at funerals constitutes a high risk because of the rites/rituals performed prior to burial including touching, washing, and dressing cholera corpses. Those who perform the rites/rituals often also serve food to funeral attendees. These pose great danger to attendees as proper handwashing is often problematic.

**Cemetery on mountains:** While KIs focused on the risk of exposure linked with eating at funerals, FGDs concentrated on mountaintop burial practices. For example: *cholera is persistent in Doualare because of the cemetery installed on the mountaintop. Because the dead are not buried at the right depth, in the rainy season, runoff contaminates wells at the foot of the mountain leading to the resurfacing of cholera every year. Mountaineers including Mofu, Matakam, and Zulgo ethnic groups, when one of them contracts cholera, they avoid hospital, hide the patient to bury the dead on mountain in case of death, which is why in the past three years cholera always starts in Doualare,* (FGD, women only group, Doualare, Maroua II).

For the ethnic groups mentioned in this excerpt, they shun public health approved cemeteries and maintain their cemetery on the mountaintop. This has been passed down from generation to generation such that at the time of death, the dying usually calls for mountaintop burial. If it is decided otherwise to bury the dead elsewhere Mofu, Matakam, and Zulgo ethnic groups hold that digging a grave elsewhere will be met with a stone; in other words, only on the mountaintop will digging a grave not encounter a stone. Ironically, mountaintops have more stones in that region, which is why corpses are not buried at the proper depth. The local council prohibits mountain burial, which, regrettably, leads to hospital avoidance, hiding cholera patients and corpses.

**Weddings:** HHDs identified that weddings pose a high risk for cholera transmission because foods come from different sources. In a typical wedding, family members of bride, groom, and friends contribute foods for the wedding. Such foods come from many sources, and are left for long hours without refrigeration, and typically served without reheating. There is hardly any means to track the conditions under which the foods were prepared. Yet, the issue of eating at weddings during cholera season elicited split opinions; some participants said they would not eat at weddings while others said they would just taste the food in order not to upset the bride and groom: *Yes, I will eat in a wedding if someone in that family was recently cured of cholera. However, if the patient died, in order not to upset the bride, groom and their family members, I will at least taste the food,* (HHD, Makabaye, Maroua I).

This quotation above illustrates how the thinking is not about healthy behavior to avoid disease. It is rather about solidarity. ‘At least taste the food’ ties with the notion of belonging to a family as we saw with group eating (‘faire un rond’).

**Open defecation:** Both KIs and FGDs identified open defecation as a behavior that promotes cholera exposure. For Zoulgo, Kole, Musgum, and Peul ethnic groups, open defecation was used to fertilize the soil for cultivation. Besides, the latter ethnic groups also believed that mixing child excrement with adult excrement in latrines leads to child stomachaches: *Open defecation fertilizes the soil. Besides, putting child excrement in the toilet gives stomachache to the child. Child excrement must be thrown around the compound. Ethnic groups with this cultural behavior include Zoulgo, Kole, Musgum, and Peul,* (FGD, men only group, Doualare, Maroua II).

Although the local council imposed a by-law on the compulsory construction of latrines, still, some were empty upon inspection. Thus, Zoulgo, Kole, Musgum, and Peul ethnic groups create the very problem they try to avoid.

### Cultural traditions that influence receptivity of public health messages

**Plurality of languages and mistrust:** Most villagers in this region speak local languages. However, during our field visits all public health messages against cholera were in French. If just a minority speaks French or English, this poses a challenge because community at large as expressed by the following extract will not understand the messages: *patients may speak one of many different local languages (Fulfulde, Kanuri, and Mandara, Hausa, Tempura, etc.) and only a minority speaks French or English. In each day, I use about three different local languages with patients. This hinders effective communication,* (KI, District Hospital-Kolofata).

Excellent interpreters and/or translators that speak the local languages are needed for effective communication of public health messages. However, getting an excellent interpreter/translator is not enough. The interpreter/translator must be one from the community in whom the inhabitants trust: *they are very receptive to well respected and trusted community health workers. One must get a trustworthy person from the community that can discern whether the person is telling the truth. If trust is lacking, they will either say they don't know or say what you want to hear,* (KI, health worker-Kolofata).

Plurality of languages and mistrust hinder the receptivity of public health messages in the Far North.

**Beliefs against pharmaceutical medicines:** Beliefs about the consequences of consuming pharmaceuticals and the healing power of traditional medicine hinders receptivity of public health messages: *there is a belief that too much consumption of pharmaceutical products destroys human organs, reduces his life span, and leads to white hair, decreases fertility and increases premature aging. As such, traditional medicine is believed to be superior. In addition, there is rural-urban mentality confrontation and the former wins because the people have more confidence in traditional medicine,* (FGD, men only group, Doualare, Maroua II).

The foregoing excerpt is an argument in defense of traditional medicines. This belief differs between FGD participants in the urban and rural areas in what was termed ‘rural-urban mentality confrontation’.

**Rural-urban mentality confrontation:** This articulates how those in rural areas trust in traditional medicine, while urban duelers believe in both traditional and pharmaceutical medicines as summed up by FGDs: *There is rural-urban mentality confrontation between family members who live in the rural areas and those in the urban cities. Cholera patients in urban areas seek care in hospitals but once family members in rural areas learn about the disease and hospitalization, they would report to the health facility with traditional medicines and insist on traditional remedies. This leads to the abandonment of not only the hospital but also the cholera treatment in favor of traditional remedies as the latter tends to come from the elderly in the village who are more trusted and always have the final say; and thus, the trust in traditional over pharmaceutical medicines,*(FGD, men only group, Doualare, Maroua II).

A religious factor further complicates the picture with Muslims preferring to copy verses from the Quran, dip them in water and offer as remedy to diseases including cholera. Still, Muslims regard Western biomedicines as products of pagans (*‘produits des païens’*) and homosexuals; and thus, consuming them is profanity (*‘Haram’* in Arabic). In addition, for Muslims, dying at home means dying with fellow Muslims who are considered ‘pure’ in contrast to dying at the hands of pagans in the hospital. All these affect public health messages receptivity because they are used to justify reluctance to seek care or abandon it. Even when the messages were received or listened to, they were not acted upon accordingly leading to what KIs and FGDs termed culture of dependency and sentimental animal husbandry, respectively.

**Culture of dependency:** Culture of dependency or *‘cultur de dépendance’* expresses the reluctance of wealthy people to contribute in kind or cash towards public goods. They firmly belief that the provision of public facilities (latrines and wells) is the responsibility of others: *to motivate some villages to collect money or contribute cattle to dig a well or latrine is very difficult. Even though they can do it, some beliefs of some of the villages (particularly Fulani-Muslim dominated areas) compel them to wait for the government or Non-Governmental Organization to provide water and sanitation facilities,* (KI, Maroua Urban).

Of note is that villages that show reluctance to contribute to the common goods would not act to maintain such facilities once they are provided to them. Although they suffer from cholera and fully aware that good facilities would prevent disease, still, change to provide and maintain facilities is difficult. Still under ‘culture of dependency’, women and children must receive permission from the head of household (the man) to seek care and complete treatments at health facilities: *women must get permission from the men to receive treatment in the hospital despite availability of free treatment. They avoid the hospital and even escape from the hospital when community health workers spot and bring them in for treatment,* (KI, cholera command and control center, Maroua Urban).

KIs illustrated this dependency of women and children by showing a picture of a nine-months-old child who was severely dehydrated, but could not get care without the father's permission. FGDs confirmed the latter citing women from Bornois ethnic groups as examples but attribution it to what they termed culture of sentimental animal husbandry.

**Culture of sentimental animal husbandry:** FGDs attributed receptivity but non-response to public health messages to culture of sentimental animal husbandry: *reluctance to contribute animals towards public facilities is because of what is called culture of sentimental animal husbandry (‘culture d´élevage sentimental’), which makes some breeders cling to their animals even when they become old and unproductive,* (FGDs, Women only group, Maroua III).

Sentimental animal husbandry expresses how some cattle breeders derive self-worth, prestige, and importance in the number and duration of cattle ownership. The higher the number of cattle and the longer the duration they keep them, the higher the breeders' self-worth, prestige, and socio-economic-status. Any reduction in numbers of cattle through say contribution to dig a latrine or well reduces self-worth, prestige and importance. Because of these, they develop strong sentiments towards their cattle that lead to the inability to relinquish them.

## Discussion

This qualitative study highlights cultural influences on cholera exposure and transmission within an urban and a rural district in FN, Cameroon. We found that KIs attributed the etiology of cholera to dirt while FGDs/HHDs attributed it to a reprimand from ‘god’. However, cholera's causative agent *Vibrio cholera* was never mentioned. Still, both KIs and FGDs/HHDs share communality by suggesting that cholera spread through water (*‘caneri’*) and food (group eating) but differ in that FGDs/HHDs believe cholera also spread through the air. A study in Guinea Bissau indexed Papal people who believe that sickness results from ritual failure; they perceive cholera as being transmitted through the air [17]. As such, a person who holds the notion that cholera is punishment from ‘god’ that spreads through the air would be more inclined to seek cholera treatment from traditional healers than health facilities, and more likely to perform traditional rituals than proper handwashing and hygiene behaviors. These delay proper treatment at health facilities leading perhaps to the high case fatality ratios observed during the 2009/10 outbreaks in the FN (Figure 1C and D). Our finding that FGD participants preferred traditional medicine, in the context of cholera treatment, over biomedicine confirms that of an ethnography on diabetes in Cameroon, which documented diabetes patients' preference of traditional over biomedicine [24]. Although traditional healers' claim to treat cholera are doubtful, still, we concur with Mbeh et al. [25] that they should be trained on cholera prevention and control measures, and to provide psychosocial support to patients [24] to counter beliefs that cholera is punishment from ‘god’ and is transmitted through the air.

From a public health standpoint, group eating [26-28] or *‘fair un rond’* poses great risks of cholera exposure but for people in the FN, it is a cultural way of life linked with feelings of belonging to a family. Funerals [19] and weddings [29] are needed, but create unique risks of cholera exposure and rapid transmission. Still, closely linked with funerals is the practice of mountain burial with the belief that burial in a recommended cemetery will be met with a stone. This is counterintuitive. The rocky nature of the mountains does not favor proper burial, let alone proper protection from rain exhuming corpses. Likewise, open defecation for soil fertilization and avoiding child stomachache are risky behaviors, which creates a vicious cycle where water and food would be contaminated through run off. The child consumes the latter leading to more child stomachache including cholera. Unfortunately, we see a culture creating the very problem it is attempting to avoid. Some of the other outstanding findings of this qualitative study relates to the receptivity but nonresponse to public health messages. In this vein, this study reveals that the fact that someone listen to or read messages about cholera prevention does not automatically translate into acting according to what the messages say. In the FN, not only do messages need to be in the local languages, but also they need to come from someone in whom the community trust. In a setting where plurality of cultural factors including mistrust in pharmaceutical products, rural-urban mentality confrontation, culture of dependency and sentimental animal husbandry lead to receptivity but nonresponse to public health messages, we agree with Panter-Brick et al. [30] and Awah [24] that public health messages should be culturally impelling and inclusive. This study has important limitations. The major limitation is the small sample size of participants studied, which makes it impossible to generalize results for the whole of Cameroon. However, in-depth data were obtained from the participants, which would not have been possible with a larger sample size. Further, in phase II, FGDs were not held in Kolofata because of insecurity linked with Boka Haram. Despite the limitations, we were able to identify factors linked to culture that could clearly influence cholera exposure and hinder awareness raising campaign messages.

## Conclusion

Throughout this qualitative study, participants acknowledged and understood cultural influences on cholera exposure and transmission, but could not avoid these influences. Accordingly, we recognize this tension between risk recognition and risk avoidance; people recognize risks but cannot afford (culturally/financially) to avoid them. Many participants had limited scientific knowledge about the cause of cholera and its transmission. The cultural practice of mountain burial may be at least one factor contributing to the high cholera attack rates in the mountainous terrain compared to the floodplains. Cultural factors are likely to play important roles in the transmission of cholera in the FN. Understanding the cultural context, beliefs, individual and community perceptions of the risk and disease may help local public health agencies and hospitals in response to outbreak prevention and/or control of transmission. This may be very critical in a setting where plurality of languages and mistrust affects receptivity of awareness campaign messages and adherence to treatment.

**Recommendations:** Cholera is an ancient disease mainly addressed through awareness raising campaigns and treatment. Though practices might not change, new behaviors could be incorporated to mitigate risks. For example, *‘caneri’* could be modernized to narrow-mouthed jars with spigots in addition to making household water chlorination affordable to all. Group eating should be encouraged, but with each person having his/her own plate. Funerals and weddings should be banned in the entire Far North during cholera season from May to October [31] and conventional cemeteries need to be encouraged in Doualare and other regions practicing mountaintop burial. Concerted education is also needed to offset open defecation as means of soil fertilization and prevention of child stomachache. In addition, culture/behavior of dependency needs to be addressed through education and economic empowerment of women. Trustworthy interpreters are needed to counter plurality of languages and mistrust of public health messages. Furthermore, trust in traditional remedies, rural-urban mentality confrontation, and culture of sentimental animal husbandry all hinder reception of public health messages against cholera and contribute to hospital avoidance, abandonment of cholera treatment, and hiding cholera patients and corpses. Due to their trust in the community and psychosocial skills, traditional healers should be included to deliver culturally inclusive messages during awareness campaigns. Appropriately designed quantitative studies to confirm the link between each of these observations and disease occurrence and spread is here called for; nonetheless, these observations serve as a starting point for hypothesis generation and initial program/intervention development.

### What is known about this topic

- Culture has the potential to influence disease exposure;
- Practices and behavior have the potential to influence disease transmission;
- The Far North Region of Cameroon is prone to cholera outbreaks.

### What this study adds

- Cultural beliefs about etiology and perceptions of spread of cholera differ depending on the sub section of the population. There is no shared understanding of the etiology of cholera between the literate and grassroots lay people of the Far North. A person that holds the notion that cholera is reprimand from God that spreads through the air would be less likely to yield to public health messages of maintaining clean water, adequate sanitation, and proper hygiene to prevent cholera. As such, public health authorities need to address the poor understanding of cholera etiology and spread at the grassroots level for these prevention strategies to be effective;
- Cultural practices that influence cholera exposure: Funerals, weddings, mountaintop burial, and open defecation were identified as cholera exposure practices. As funerals and wedding practices bring large number of people together at a given time, these favor rapid transmission of cholera; and thus, suggest that these feast should be banned during cholera season. As regards the latter two findings, the practice of burying corpses on mountain contributes to hiding cholera patients and avoiding health facilities while open defecation leads to contamination of water sources and disease transmission through flies. These could be changed through targeted and sustained education;
- To the cultural traditions that influence or act as barriers to receptivity of public health prevention messages against cholera were plurality of languages and mistrust, superiority of traditional over pharmaceutical medicines (rural-urban mentality confrontation), culture of dependency and sentimental animal husbandry. To address these points, trust worthy community health workers (including traditional healers) with knowledge of local languages should be deployed to communicate culturally impelling messages for the fight against cholera during awareness raising campaigns.

## Competing interests

The authors declare no competing interests.
